# Beyond base camp: PI3K/mTOR inhibition for the treatment of pediatric high-grade gliomas

**DOI:** 10.1093/neuonc/noaf163

**Published:** 2025-09-11

**Authors:** Ryan J Duchatel, Clara Savary, Zacary P Germon, Madisen Riley, David S Ziegler, Sabine Mueller, Evangeline R Jackson, Matthew D Dun

**Affiliations:** Paediatric Stream, Mark Hughes Foundation Centre for Brain Cancer Research, College of Health, Medicine and Wellbeing, University of Newcastle, Callaghan, New South Wales, Australia; Precision Medicine Research Program, Hunter Medical Research Institute, New Lambton Heights, New South Wales, Australia; Cancer Signalling Research Group, School of Biomedical Science and Pharmacy, College of Health, Medicine and Wellbeing, University of Newcastle, Callaghan, New South Wales, Australia; Institut des Sciences Exactes et Appliquées, Université de la Nouvelle-Calédonie, Nouméa, New Caledonia; Paediatric Stream, Mark Hughes Foundation Centre for Brain Cancer Research, College of Health, Medicine and Wellbeing, University of Newcastle, Callaghan, New South Wales, Australia; Precision Medicine Research Program, Hunter Medical Research Institute, New Lambton Heights, New South Wales, Australia; Cancer Signalling Research Group, School of Biomedical Science and Pharmacy, College of Health, Medicine and Wellbeing, University of Newcastle, Callaghan, New South Wales, Australia; Paediatric Stream, Mark Hughes Foundation Centre for Brain Cancer Research, College of Health, Medicine and Wellbeing, University of Newcastle, Callaghan, New South Wales, Australia; Precision Medicine Research Program, Hunter Medical Research Institute, New Lambton Heights, New South Wales, Australia; Cancer Signalling Research Group, School of Biomedical Science and Pharmacy, College of Health, Medicine and Wellbeing, University of Newcastle, Callaghan, New South Wales, Australia; Paediatric Stream, Mark Hughes Foundation Centre for Brain Cancer Research, College of Health, Medicine and Wellbeing, University of Newcastle, Callaghan, New South Wales, Australia; Precision Medicine Research Program, Hunter Medical Research Institute, New Lambton Heights, New South Wales, Australia; Cancer Signalling Research Group, School of Biomedical Science and Pharmacy, College of Health, Medicine and Wellbeing, University of Newcastle, Callaghan, New South Wales, Australia; Kid’s Cancer Centre, Sydney Children’s Hospital, Randwick, New South Wales, Australia; School of Clinical Medicine, University of New South Wales, Sydney, New South Wales, Australia; Children’s Cancer Institute, Lowy Cancer Research Centre, University of New South Wales, Sydney, New South Wales, Australia; Department of Pediatrics, University of Zurich, Zurich, Switzerland; Department of Neurology, Neurosurgery, and Pediatrics, University of California, San Francisco, San Francisco, California, USA; Paediatric Stream, Mark Hughes Foundation Centre for Brain Cancer Research, College of Health, Medicine and Wellbeing, University of Newcastle, Callaghan, New South Wales, Australia; Precision Medicine Research Program, Hunter Medical Research Institute, New Lambton Heights, New South Wales, Australia; Cancer Signalling Research Group, School of Biomedical Science and Pharmacy, College of Health, Medicine and Wellbeing, University of Newcastle, Callaghan, New South Wales, Australia; Paediatric Stream, Mark Hughes Foundation Centre for Brain Cancer Research, College of Health, Medicine and Wellbeing, University of Newcastle, Callaghan, New South Wales, Australia; Precision Medicine Research Program, Hunter Medical Research Institute, New Lambton Heights, New South Wales, Australia; Cancer Signalling Research Group, School of Biomedical Science and Pharmacy, College of Health, Medicine and Wellbeing, University of Newcastle, Callaghan, New South Wales, Australia

**Keywords:** PI3K, mTOR, diffuse midline glioma, clinical trials, high-grade glioma

## Abstract

Pediatric high-grade glioma (pHGG), including diffuse midline glioma (DMG), are the most aggressive and fatal pediatric cancers. Mutations and amplifications within the phosphatidylinositol-4,5-bisphosphate 3-kinase (PI3K) pathway drive tumor growth, treatment resistance, and poor outcomes. Although PI3K and mTOR have been identified as genetic dependencies in pHGGs, translating this knowledge into effective treatment remains challenging. The blood–brain barrier (BBB) restricts the delivery of most PI3K/mTOR inhibitors and , hence, often show poor CNS penetration. Even when present in the brain, these agents frequently encounter adaptive resistance mechanisms that blunt efficacy. Side effects, including hyperglycemia, rash, and mucositis, further complicate their use and reduce compliance. Encouragingly, novel brain-penetrant PI3K/mTOR inhibitors offer new opportunities for treatment, but combining these agents with other therapies, including chemotherapy, other small molecules, and immunotherapies, requires careful balancing of toxicity and efficacy. Therefore, achieving optimal dosing for each patient remains a significant hurdle. This review examines the promise and pitfalls of targeting the PI3K/mTOR pathway in DMG, including the limitations of available therapies, mechanisms of resistance, and the critical need for improved regimen design. We propose a roadmap to guide future efforts, emphasizing rational combination strategies and better patient stratification to improve survival for children diagnosed with these devastating cancers.

**Key Points**

- High-grade gliomas (HGGs), including diffuse midline glioma (DMG), show a dependency on the PI3K/mTOR pathway, with patients harboring recurring genetic alterations.
- Brain-penetrant PI3K/mTOR offers hope for improved precision-guided treatment.
- Children are not adults; clinical trials have overlooked key limitations of PI3K/mTOR inhibition in pediatric brain tumors.

## Background

### Opportunities and Challenges in Pediatric High-Grade Gliomas

Diffuse midline glioma (DMG) is the most common and lethal pediatric high-grade glioma (HGG), with a universally fatal prognosis. Median overall survival ranges from just 9 to 15 months,^1^ with children diagnosed with diffuse lesions in the pontine region of the brainstem, termed diffuse intrinsic pontine glioma (DIPG), typically succumbing within a year of diagnosis. Palliative radiotherapy (RT) remains the only standard of care outside clinical trials. Reirradiation is increasingly used in longer-term survivors but offers only a modest survival benefit (4.2–6.9 months),^2,3^ underscoring the urgent need for effective therapeutic strategies. Mutations and amplifications in signaling genes *PDGFRA*, *PIK3CA/PIK3R1*, *FGFR*, *EGFR*, and *ACVR1* coupled with the loss of function of tumor suppressor genes *TP53* and *PTEN* combine with hallmark instigating H3-alterations (*H3-3A*, *H3C2/H3C3*, *EZHIP*) to drive a highly clonal disease, limiting the benefit of single agents.^4,5^ In the absence of specific H3K27M-targeted therapies, pharmacological inhibition of overexpressed or constitutively activated signaling pathways remains a key therapeutic strategy under intensive investigation for the treatment of DIPG/DMG. However, past clinical trials for DMG have failed to improve survival, underscoring the urgent need for more effective therapeutic options. One of the major challenges in treating DMG is the blood–brain barrier (BBB), which significantly restricts drug delivery.^6^ HGGs originating in the midline, such as DMGs, exhibit even lower BBB permeability (“leakiness”) than other HGGs, including glioblastomas,^7^ further complicating efforts to develop pharmacological agents capable of improving patient outcomes.

## PI3K/mTOR Signaling in DMG: A Key Driver and Therapeutic Opportunity

**Figure 1.**
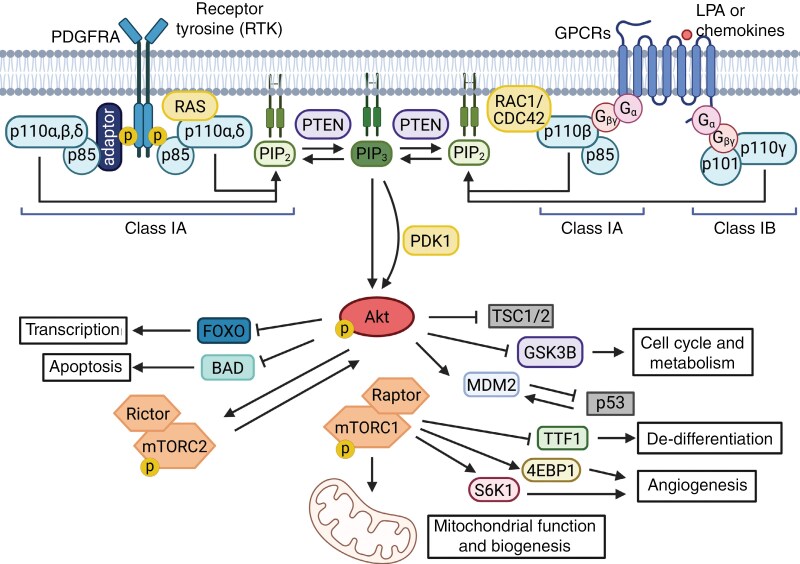
Signal transduction via PI3K/Akt/mTOR pathway promoting brain cancer growth and survival. The phosphatidylinositol 3-kinase (PI3K) pathway (light blue) is activated by receptor tyrosine kinases (RTKs) or G-protein–coupled receptors (GPCRs) upon binding of growth factors (eg, PDGFRA, VEGF) or cytokines. This activation leads to the phosphorylation of phosphatidylinositol-4,5-bisphosphate (PIP₂, light green) to generate phosphatidylinositol-3,4,5-trisphosphate (PIP₃, dark green). PIP₃ recruits pleckstrin homology (PH) domain-containing proteins, including Akt (red), to the membrane, triggering downstream signaling cascades. Akt phosphorylates multiple targets to regulate key oncogenic processes such as transcription, cell cycle progression, metabolism, and apoptosis. Downstream, mTOR complex 1 (mTORC1, orange) promotes tumorigenesis by modulating autophagy, angiogenesis, and cellular de-differentiation. The tumor suppressor PTEN (purple) negatively regulates the pathway by dephosphorylating PIP₃ to PIP₂. Further regulation occurs through inhibition of tumor suppressor proteins, including p53 and the TSC complex (both shown in gray), contributing to uncontrolled cell growth and survival in brain tumors. (Created in BioRender. Dun, M. (2025) https://BioRender.com/fp6r4ob, adapted from^4,12^).

One of the most important contributions to the malignant growth, metabolism, survival, and migration of HGGs is the phosphatidylinositol 3-kinase (PI3K) lipid kinase signaling pathway.^8,9^ The PI3K gene family is divided into 3 PI3K classes (I–III) based upon their different structures and substrates.^10^ Functionally, the PI3K family is comprised of the catalytic subunit p110α (*PIK3CA*) and a regulatory subunit p85α (*PIK3R1*—including regulatory isoforms p55 and p50).^11^ Class I PI3Ks form a heterodimeric complex comprised 1 of 4 catalytic isoforms—p110α (*PIK3CA*), p110β (*PIK3CB*), p110δ (*PIK3CD*), p110γ (*PIK3CG*)—and a regulatory isoform, which may be p85α, p55α, p50α (all encoded by *PIK3R1*), p85β (*PIK3R2*), p55γ (*PIK3R3*), p101 (*PIK3R5*), p84, or p87 (*PIK3R6*).^11^ This class of PI3Ks is commonly found mutated in cancers, particularly p110α/*PIK3*CA. Upon activation, PI3K catalyzes the conversion of PIP2 to PIP3, a second messenger that recruits Akt to the membrane, where it becomes activated. Akt, in turn, activates numerous downstream targets that promote cell growth, survival, and metabolism (Figure 1). Alterations in platelet-derived growth factor receptor alpha (*PDGFRA*) are also a frequent genomic event in DMG and HGG and are strongly associated with the H3.3K27M mutations (30%) and drive downstream signaling through the PI3K/Akt/mTOR pathway.^4,5^ In addition, activating mutations are seen in *PIK3CA* (12%) across all H3 subtypes, *PIK3R1* (18%) in H3.3K27M and EZHIP subtypes, *TSC2* (2%) in EZHIP subtypes, *RPTOR* (1%) only in H3.3K27M subtypes, as well as *MTOR* (1%) in H3.3K27M (34), while loss of function of the tumor suppressor protein *PTEN*, the negative regulator of this pathway is observed in 19% of DMG cases.^4,5^ We recently analyzed the functional consequence of PI3K expression across 38 patient-derived DMG models encompassing all H3-subtypes,^13^ using a CRISPR/Cas9 loss-of-function screening approach. This demonstrated that the *PIK3CA* catalytic subunit of PI3K/Akt/mTOR signaling, as well as MTORC1, are genetic dependencies, independent of activating mutations in any of the PI3K subunits (*PIK3CA*/*PIK3R1/AKT/MTOR)*. These findings were confirmed by the knockout of *PTEN*, increasing growth.^13^ Targeted deletion of *PIK3CA* (CRISPR-Cas9) in a PI3K/mTOR wildtype H3.3K27M DMG cell line (SU-DIPG-XIII) completely ablated in vitro proliferation, highlighting the importance of PI3K signaling in DMG regardless of the presence of activating mutations in PI3K-related genes.^13^

This may be explained by several studies suggesting that phosphatidylinositol (PI) metabolism is upregulated in HGGs, contributing to constitutive activation of PI3K signaling and tumor progression. The PI3K/Akt/mTOR pathway, driven by PI-derived phosphoinositides, is frequently hyperactivated in HGGs due to mutations/alterations leading to elevated PIP3 levels and downstream Akt/mTOR activation.^14,15^ Metabolomic studies have demonstrated that these cancers exhibit altered lipid profiles, including increased levels of PIs, which may facilitate enhanced PI3K activity independent of activating mutation.^16^ Moreover, the higher the tumor grade, the more increased the PI3K pathway is, correlating with poor prognosis and therapy resistance.^16^ Elevated PI levels may act as a catabolic driver of constitutive PI3K signaling, fueling glioma progression and metabolic adaptation, offering new targets for combination strategies.

Additional elegant studies have hypothesized that targeting PI3K/Akt/mTOR signaling is critical to improve the outcomes of cancer patients. More than 10 different clinical trials have evaluated the efficacy of compounds targeting PI3K/Akt or mTOR for the treatment of DMG (Table 1); unfortunately, none are yet to progress further than safety and early efficacy studies. Importantly, early-stage clinical trial data in other cancers suggest that targeting co-activated pathways with combination inhibitors is more effective than monotherapeutic approaches, owing to the simultaneous inhibition of intrinsic and adaptive resistance mechanisms, further explored in Section “Resistance to PI3K/mTOR inhibition in DMG”.^23,24^

**Table 1.** Overview of Clinical Trials Investigating Pan-PI3K Inhibitors in Central Nervous System-Related Cancers

Drug	PI3K type	Cancer type	Adjuvant	Common side effects	Dose/half-life	Phase/approval
Paxalisib (GDC-0084)	Pan-PI3K	DIPG/DMG	None	Rash (5/16) Neutropenia (4/16) Hyperglycemia (2/16)	MTD (Human) = 27 mg/m^2^/d Half-life (Human) = 20.6 ± 9.1 h^17^	Phase I—Completed (NCT03696355)
Paxalisib (GDC-0084)	Pan-PI3K	Progressive or recurrent HGG	None	Fatigue (30%) Hyperglycemia (28%) Nausea (23%) Rash (17%) Hypertriglyceridemia (15%) Mucositis (15%) Hypophosphatemia (13%) Decreased appetite (11%) Diarrhea (11%)	MTD (Human) = 45 mg/d,^18^ Half-life (Human) = unknown	Phase I—Completed (NCT01547546)
Paxalisib (GDC-0084)	Pan-PI3K	DIPG/DMG	ONC201 and radiation therapy	No results posted	Dosing regimens not posted	Phase II—Active (NCT05009992)
Paxalisib (GDC-0084)	Pan-PI3K	Glioblastoma	Unmethylated MGMT promoter status following surgical resection and initial chemoradiation with temozolomide	No results posted	Dose (Human) = 60 mg initially, future dose cohorts = received 15 mg increasingly until tolerated Half-life (Human) = unknown	Phase II—Completed (NCT03522298)
Paxalisib (GDC-0084)	Pan-PI3K	Glioblastoma	Metformin and ketogenic diet	No results posted	Dose (Human) = 45 mg/d of paxalisib initially, if tolerated dose will increase to 60 mg/d after 28 days Half-life (Human) = unknown Dose (Human) = 850 mg of metformin as initial dose for cycle 1, day 1. Dose will increase to 1700 mg/d on cycle 2, will be further increased to 2550 mg/d on cycle 3 Ketogenic diet will be maintained throughout trial	Phase II—Recruiting (NCT05183204)
Paxalisib (GDC-0084)	Pan-PI3K	Glioblastoma	None	No results posted	Dose (Human) = 5 mg of paxalisib every day for 28 days for cycle 1. If tolerated dose will increase to 60 mg every day for 28 days Half-life (Human) = unknown	Phase II and III—Recruiting (NCT03970447)
Paxalisib (GDC-0084)	Pan-PI3K	Solid tumors—brain metastases	None (Experimental arm II)	No results posted	Dose regimen not posted	Phase II—Recruiting (NCT03994796)
Paxalisib (GDC-0084)	Pan-PI3K	Pediatric solid tumors	Irinotecan and temozolomide	No results posted	Dose (Human) = 50 mg/d of irinotecan intravenously on days 1–5, 28-day cycle, 13 cycles Dose (Human) = 150 mg/d of temozolomide, orally, on days 1–5, 28-day cycle, 13 cycles Dose (Human) = 21 mg/daily of paxalisib, orally, 28-day cycle, 13 cycles Half-life (Human) = unknown	Phase I/II—Recruiting (NCT06208657) (OPTIMIZE)
Paxalisib (GDC-0084)	Pan-PI3K	Recurrent or refractory primary central nervous system lymphoma	None	No results posted	Dose not posted. Cycles will last up to 28 days for 24 months	Phase II—Recruiting (NCT04906096)
Buparlisib (BKM-120)	Pan-PI3K	Glioblastoma	Surgery (cohort 1) None (cohort 2)	Lipase elevation (10.8%) Fatigue (6.2%) Hyperglycemia (4.6%) Elevated alanine transaminase (4.6%)	Dose (Human) = 100 mg once daily, for 8–12 days prior to surgery. After surgery, 100 mg once daily was given for 28-day cycles Dose (Human) = 100 mg once daily, for 28 cycles (nonsurgical cohort) Half-life = approx. 40 h^19^	Phase II—Completed (NCT01339052)
Buparlisib (BKM-120)	Pan-PI3K	Glioblastoma	INC280	Fatigue (36.4%) Nausea (30.3%) Increased alanine aminotransferase (30.3%) Aspartate aminotransferase increased (24.2%) Depression (24.2%) Hyperglycemia (21.2%)	Dose (Human) = 200 mg of INC280 initially, twice daily with dose escalations in phase Ib Dose (Human) = 400 mg of INC280 twice daily in phase II Dose (Human) = 50 mg of buparlisib once daily as an initial dose with escalation of doses after^20^ Half-life (Human) = unknown	Phase Ib and II—Terminated (NCT01870726)
Buparlisib (BKM-120)	Pan-PI3K	Glioblastoma	Temozolomide and radiation therapy	*Stage II: Concomitant + adjuvant* Nausea (56.3%) Fatigue (56.3%) Decreased lymphocyte count (31.3%) Thrombocytopenia (31.3%) Decreased neutrophil count (31.3%) Hyperglycemia (12.5%) Increased amylase (12.5%) *Stage I: Adjuvant* Nausea (72.7%) Fatigue (59.1%) Hyperglycemia (18.2%) Thrombocytopenia (18.2%) Decreased platelet count (13.6%) Cognitive disorder (13.6%) Confusional state (13.6%)	Dose (Human) = daily dose of buparlisib at 40, 60, 80, or 100 mg in combination with temozolomide at 5, 20, 100, 140, 180, or 250 mg^21^ Half-life (Human) = unknown	Phase I—Completed (NCT01473901)
Buparlisib (BKM-120)	Pan-PI3K	Glioblastoma	Bevacizumab	Fatigue (37%) Hyperglycemia (26%) Increased alanine transaminase (25%)	Dose (Human) = once daily at 60 or 80 mg of buparlisib and 10 mg/kg intravenously every 2 weeks of bevacizumab ^22^ Half-life (Human) = unknown	Phase I and II—Completed (NCT01349660)
Buparlisib (BKM-120)	Pan-PI3K	Breast cancer—brain metastases	Capecitabine	Nausea (6/10) (60%) Fatigue (5/10) (50%) Hypokalemia (5/10) 50%) Anorexia (5/10) 50%) Mucositis (4/10) 40%) Diarrhea (4/10) 40%) Urinary tract infection (2/10) (20%) Seizure (2/10) (20%)	Dose (Human) = 100 mg of buparlisib daily with capecitabine at a dose of 1000 mg twice a day—14 days on and 7 days off Half-life (Human) = unknown	Phase II—Completed (NCT02000882)
Buparlisib (BKM-120)	Pan-PI3K	Melanoma—brain metastases	None	No results posted	Dose (Human) = 100 mg orally, once daily	Phase II—Unknown status (NCT02452294)

Abbreviations: DIPG, diffuse intrinsic pontine glioma; DMG, diffuse midline glioma; HGG, high-grade glioma; MTD, maximum tolerated dose.

Summary of ongoing and completed clinical trials evaluating the pan-PI3K inhibitors paxalisib and buparlisib in central nervous system (CNS) malignancies. Included are the cancer types, clinical trial phases, common treatment-related side effects, dosing regimens, and reported half-lives for each agent.

### The Role of PI3K Signaling in Neurodevelopment and Disease

PI3K signaling is essential for neurodevelopment, particularly during embryogenesis, where trophic factors such as Insulin-like Growth Factor-1 (IGF1) play a critical role.^25^ PI3K is fundamental for normal brain size and function, with mutations in *PIK3CA* during embryonic development leading to severe overgrowth disorders known as PIK3CA-related overgrowth spectrum (PROS).^26^ These disorders include bilateral dysplastic megalencephaly, hemi-megalencephaly, and focal cortical dysplasia, the latter being a major cause of intractable pediatric epilepsy.^27^ Neuronal stem cells in the developing brainstem rely heavily on PI3K signaling, including regulation by PTEN, underscoring the pathway’s dual role: essential for survival, and capable of causing severe abnormalities when dysregulated.

Common *PIK3CA* hotspot mutations seen in children with brain overgrowth disorders are also found as obligate partners to H3K27M mutations in HGGs and DMG, including H1047R and E545K.^28^ Mouse models expressing the most common activating mutations in *Pik3ca* (H1047R and E545K) induced at embryonic day (E0.5), displayed progressive hydrocephalus, ventriculomegaly, and megalencephaly, leading to death before weaning. However, when the same mutations were activated postnatally, they did not cause PROS, suggesting that *PIK3CA*-driven overgrowth disorders arise only when mutations occur in embryonic neural progenitors.^28^ Whether this same principle applies to *PIK3CA* mutations in HGGs remains unknown. Indeed, postnatal mice with activated *Pik3ca* transgenes responded to acute treatment with BKM-120 (buparlisib—discussed in Section: Pan-PI3K Inhibitors in Clinical Development: Paxalisib and Buparlisib), a brain-penetrant pan-PI3K inhibitor, showing significant antiepileptic effects.^29^ These findings suggest that PI3K inhibitors could offer a promising new approach for managing intractable pediatric epilepsy associated with PIK3CA-driven disorders.

### Clinical Trials Targeting PI3K/mTOR Signaling in DMG

**Figure 2.**
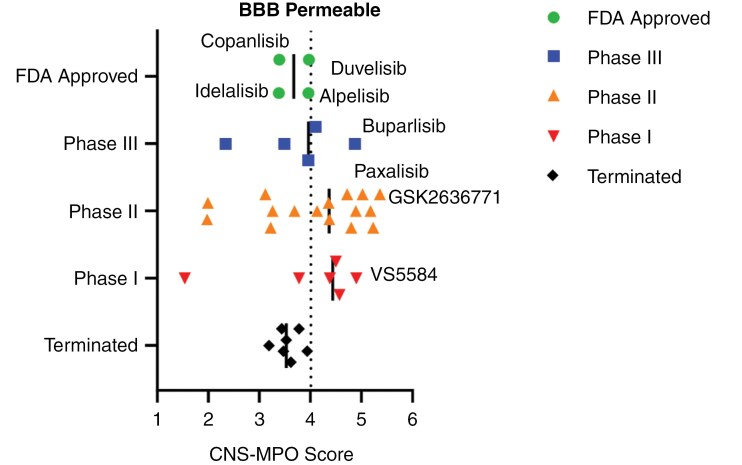
Clinical development of blood–brain barrier(BBB)-penetrant PI3K inhibitors. PI3K inhibitors currently in clinical development are shown, categorized by their clinical trial phase, and assessed for predicted BBB penetration using the Central Nervous System–Multi-parameter Optimization (CNS-MPO)^40^ scoring system. Compounds with CNS-MPO scores ≥4 are considered likely to penetrate the BBB, supporting their potential utility in treating brain tumors such as pediatric high-grade gliomas (HGGs) and diffuse midline glioma (DMG).

The *PIK3CA* gene is also mutated in 18% of breast cancers,^30^ 39% of endometrial,^31^ and 9% of nonsmall-cell lung cancer,^32^ highlighting it as a key therapeutic focus for cancer treatment across almost all types.^10^ Despite more than 40 inhibitors undergoing preclinical and clinical development, only a few have received FDA approval as anticancer therapies, including the pan-PI3K inhibitor copanlisib,^33^ p110α (*PIK3CA*) inhibitor alpelisib,^34,35^ p110γ/δ (*PIK3CY/PIK3CD*) inhibitor duvelisib,^36^ p110δ (*PIK3CD*) inhibitors idelalisib and umbralisib.^36,37^ However, for some (duvelisib, idelalisib, and umbralisib), accelerated approvals have been withdrawn.^33^ Among mTOR inhibitors, mTOR class 1 inhibitors everolimus^38^ and temisrolimus^39^ have gained FDA approval. The limited number of approved inhibitors for clinical application can be attributed to toxicities associated with PI3K/mTOR inhibitors and their limited activity in the CNS, particularly in the context of brain tumors (Figure 2), and the development of resistance. Many of these inhibitors are not approved for brain cancers, due to their low brain penetration as demonstrated by Central Nervous System—Multi-parameter Optimization (CNS-MPO) simulation results, which can predict CNS activity (Table 2). Therefore, the design and synthesis of new BBB-penetrant PI3K/mTOR inhibitors has been a focus for DMG treatment.^13^

**Table 2.** Predicted BBB penetration potential of FDA-approved PI3K/mTOR inhibitors as determined by Central Nervous System–Multi-parameter Optimization.^40^

Drug	Copanlisib		Alpelisib		Duvelisib		Idelalisib		Umbralisib		Everolimus		Temsirolimus	
Parameter	Actual	Score	Actual	Score	Actual	Score	Actual	Score	Actual	Score	Actual	Score	Actual	Score
LOGP	-0.71	1.00	2.81	1.00	3.63	0.69	3.32	0.84	6.13	0.00	7.40	0.00	7.13	0.00
LOGD	-1.02	1.00	2.67	0.66	3.63	0.19	3.32	0.34	6.13	0.00	7.40	0.00	7.13	0.00
MW	480.53	0.14	441.47	0.42	416.87	0.59	415.43	0.60	571.56	0.00	958.24	0.00	1030.30	0.00
TPSA	139.79	0.00	101.21	0.63	86.80	1.00	99.16	0.69	105.15	0.49	204.66	0.00	241.96	0.00
HBD	3.00	0.25	3.00	0.25	2.00	0.50	2.00	0.50	2.00	0.50	3.00	0.25	4.00	0.00
PKA	7.43	1.00	2.81	1.00	4.46	1.00	3.71	1.00	5.11	1.00	0.95	1.00	0.95	1.00
CNS-MPO score	3.39 / 6		3.96 / 6		3.97 / 6		3.98 / 6		1.99 / 6		1.25 / 6		1.00 / 6	

Abbreviations: CNS-MPO, Central Nervous System–Multi-parameter Optimization; HBD, hydrogen bond donors; LogD, log of the distribution coefficient; LogP, log of the partition coefficient; MW, molecular weight; PKA, OST Basic Center; TPSA, topological polar surface area.

Assessment of approved PI3K/mTOR inhibitors for brain tumor treatment suitability using the Central Nervous System Multi-parameter Optimization (CNS-MPO) scoring system. The system evaluates six physicochemical properties to predict blood–brain barrier (BBB) penetration, with a maximum score of 6. Scores ≥4 suggest likely CNS penetration; scores <4 suggest limited CNS access.

#### Brain Penetration of PI3K/mTOR Inhibitors

To evaluate the potential of FDA-approved PI3K inhibitors for DMG, we applied the CNS-MPO scoring system. This system assesses 6 key physicochemical properties that influence a drug’s ability to penetrate the BBB: calculated partition coefficient (ClogP), distribution coefficient (ClogD) at pH 7.4, molecular weight, topological polar surface area, number of hydrogen bond donors, and the most basic center (pKa).^40^

Among FDA-approved PI3K inhibitors, copanlisib (CNS-MPO: 3.39), alpelisib (CNS-MPO: 3.96), duvelisib (CNS-MPO: 3.97), idelalisib (CNS-MPO: 3.98), and umbralisib (CNS-MPO: 1.99) suggest limited brain penetration (Table 2). Approved mTOR inhibitors scored even lower for both everolimus (CNS-MPO: 1.25) and temsirolimus (CNS-MPO: 1.00), suggesting that currently approved drugs have more active mechanisms of brain uptake, or are unlikely to be effective for brain tumors. However, there is an emerging trend of PI3K inhibitors in earlier phase clinical development, with increased brain penetration (Figure 2).

Newer PI3K/mTOR inhibitors demonstrate a more favorable CNS-MPO score: paxalisib (CNS-MPO: 4.44), buparlisib (CNS-MPO: 4.87), compound 7 (CNS-MPO: 4.72), and GCT-007 (CNS-MPO: 4.28) (Table 3). These findings suggest that while existing FDA-approved PI3K/mTOR inhibitors may have limited efficacy in HGG/DMG, next-generation compounds offer greater promise for BBB penetration and therapeutic potential.

**Table 3.** Predicted BBB penetration potential of novel PI3K/mTOR inhibitors as determined by Central Nervous System–Multi-parameter Optimization.^40^

Drug	Paxalisib		Buparlisib		Compound 7		GCT-007	
Parameter	Actual	Score	Actual	Score	Actual	Score	Actual	Score
LOGP	1.32	1.00	2.56	1.00	3.16	0.92	0.24	1.00
LOGD	1.32	1.00	2.55	0.73	3.16	0.42	0.24	1.00
MW	382.43	0.84	410.40	0.64	401.49	0.70	391.45	0.78
TPSA	117.10	0.10	89.63	1.00	92.29	0.92	120.53	0.00
HBD	2.00	0.50	2.00	0.50	1.00	0.75	2.00	0.50
PKA	2.73	1.00	5.94	1.00	0.92	1.00	2.70	1.00
CNS-MPO Score	4.44 / 6		4.87 / 6		4.72 / 6		4.28 / 6	

Abbreviations: CNS-MPO, Central Nervous System–Multiparameter Optimization; HBD, hydrogen bond donors; LogD, log of the distribution coefficient; LogP, log of the partition coefficient; MW, molecular weight; PKA, most basic center; TPSA, topological polar surface area.

CNS-MPO scores for clinical-stage PI3K inhibitors (paxalisib, buparlisib) and emerging agents (GCT-007, a PI3K inhibitor; and compound 7, an mTOR inhibitor). The CNS-MPO system evaluates six physicochemical parameters to generate a composite score (maximum = 6). Scores ≥4 indicate likely blood–brain barrier (BBB) penetration, while scores <4 suggest poor CNS access.

#### Hyperglycemia Linked to pan-PI3K inhibition

Hyperglycemia remains a significant challenge in the clinical application of PI3K inhibitors due to the PI3K/Akt pathway’s role in insulin-mediated glucose uptake.^41^ Inhibiting this pathway disrupts glucose homeostasis, leading to circulating insulin and blood glucose. The effect is exacerbated using corticosteroids such as dexamethasone to manage peritumoral inflammation and hydrocephalus, further driving hyperglycemia and limiting the clinical benefit of PI3K inhibitors. Managing hyperglycemia and optimizing dosing strategies are critical for maximizing therapeutic efficacy while minimizing toxicity.

**Figure 3.**
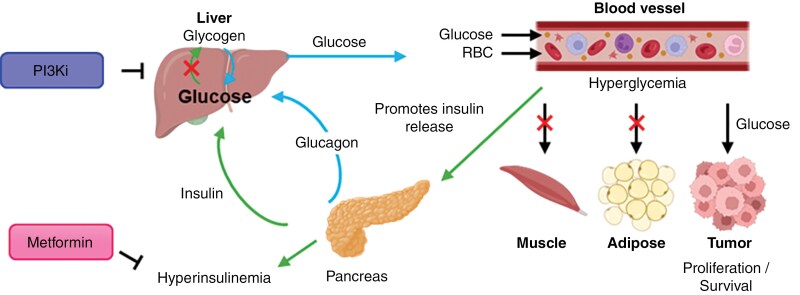
PI3K inhibition induces hyperglycemia and compensatory hyperinsulinemia. Inhibition of PI3K disrupts insulin signaling, impairing glucose uptake in insulin-sensitive tissues (eg, muscle, adipose) and reducing hepatic glycogen synthesis. This leads to systemic hyperglycemia, which may paradoxically fuel tumor growth via enhanced glucose uptake in tumors overexpressing glucose transporters. Elevated glucose levels trigger compensatory insulin secretion, resulting in hyperinsulinemia. This metabolic feedback loop can be modulated by insulin-sensitizing agents such as metformin, which reduces hepatic gluconeogenesis and improves insulin sensitivity, potentially mitigating the adverse effects of PI3K inhibition. (Created in BioRender. Dun (2025) https://BioRender.com/fp6r4ob, adapted from ^13,44,45^).

PI3K/Akt signaling, particularly downregulating AKT2, facilitates insulin-driven glucose uptake in muscle, liver, and fat cells by promoting the glucose transporters’ translation to the plasma membrane.^42^ Inhibiting PI3K blocks this process, resulting in a dose-dependent increase in plasma fasting C-peptide and insulin, leading to systemic hyperglycemia.^43^ This systemic insulin response reactivates PI3K/Akt signaling via insulin receptors, particularly in tumors with high-level insulin receptor expression, thereby limiting the effectiveness of PI3K inhibitors (Figure 3).^42^

The power of combined glycemic control using oral anti-hyperglycemia medications and PI3K inhibitors was shown in the key study “SOLAR-1” of alpelisib in patients with *PIK3CA*-mutated hormone receptor–positive breast cancer, which led to improved progression-free survival and the FDA approval of alpelisib.^34^ In this trial, 63.7% of alpelisib-treated patients experienced hyperglycemia of any grade, with 36.6%, experiencing grade 3–4 hyperglycemia, with the discontinuation rate due to hyperglycemia of 6.3%.^46^ For patients experiencing any grade of hyperglycemia, metformin was the first-line anti-diabetic agent used to manage alpelisib-induced hyperglycemia, prescribed in 76% of affected patients either alone or in combination with other anti-diabetic medications.

#### Resistance to PI3K/mTOR inhibition in DMG

Although PI3K inhibitors show promise in DMG, efficacy is often limited by compensatory pathway activation. Combining PI3K inhibition with agents targeting complementary oncogenic signals has improved tumor suppression, prolonged survival, and reduced invasion in preclinical models, offering a strategy to overcome resistance.

The combination of PI3K/mTOR inhibition (paxalisib) with MEK inhibition (mirdametinib) has shown synergistic efficacy in DMG patient-derived orthotopic xenograft models, where the combination significantly extended survival, whereas monotherapies did not.^46^ Dual treatment more effectively inhibited both pathways in the brain and reduced MAPK signaling compared to mirdametinib alone, indicating disruption of compensatory resistance mechanisms. However, these studies did not assess hyperglycemia or evaluate co-treatment with metformin or optimized dosing strategies described in Section “Hyperglycemia Linked to pan-PI3K inhibition”.

Despite the promise of PI3K inhibitors, efficacy in DMG is constrained by adaptive resistance mechanisms that preserve tumor proliferation and invasion. Protein kinase C (PKC) signaling, central to survival, migration, and progression, is a key bypass pathway. Our phosphoproteomic analyses reveal that PI3K inhibition with paxalisib increases PKC activity, where combining paxalisib with the PKC inhibitor enzastaurin showed strong efficacy in DMG models.^13^

BDNF/NTRK2 signaling activates Ras/ERK and PI3K pathways, generating IP₃ and DAG to elevate intracellular Ca²⁺ and activate PKC, physiological mechanisms co-opted by DMG cells to enhance survival and invasion.^47,48^ We showed that paxalisib elevates Ca²⁺ levels and potentiates PKC activity, promoting migration and invasion. Co-treatment with the Ca²⁺ chelator BAPTA-AM abolished this effect, suppressing phosphorylation of p-AKT, p-PKC substrates, and p-MARCKS, and significantly reducing invasion.^13^

Hyperglycemia, a common consequence of PI3K inhibition, further activates PKC via increased DAG production, contributing to vascular dysfunction, angiogenesis, and tumor progression.^49^ In HGG/DMG, corticosteroid use may amplify this effect. Our studies confirm that systemic hyperglycemia induced by PI3K inhibition at MTD correlates with enhanced PKC signaling and reduced efficacy. Moreover, PKC/MARCKS regulate adhesion, matrix remodeling, and apoptosis resistance, as shown by increased phosphorylation and invasiveness following PI3K/mTOR inhibition (paxalisib/rapamycin) or PKC activation (PMA), consistent with neuronal stimulation models.^50–52^

OPTIMISE is a molecularly stratified Phase I/II platform trial (NCT06208657), with patients enrolled on different arms based on the molecular profile of their tumor. Arm A tests the combination of paxalisib with chemotherapy (irinotecan and temozolomide [TMZ])—initially as a Phase 1 dose-escalation trial, and then expanding into Phase 2 cohorts in CNS and solid tumors. Patients in the Phase 1 cohort are not molecularly selected, but patients in Phase 2 cohorts require demonstrated activation of the PI3K/mTOR pathway for progressive pHGGs, DMG, and DIPG (Table 1). Chemotherapy resistance in these tumors is frequently driven by robust DNA damage repair and an adaptive tumor microenvironment. PI3K/mTOR signaling supports both homologous recombination and non-homologous end joining, key pathways that repair genotoxic damage from agents like TMZ and irinotecan.^53–56^ Paxalisib aims to inhibit these repair mechanisms, sensitizing tumor cells to chemotherapy and overcoming resistance.

DMG cells exhibit high metabolic plasticity, enabling adaptation to the fluctuating demands of rapid tumor growth and treatment-induced stress. As the PI3K pathway regulates key metabolic processes, this adaptability contributes to therapy resistance. Targeting mitochondrial function, specifically through activation of the mitochondrial protease ClpP, has emerged as a strategy to disrupt tumor metabolism and induce mitochondrial dysfunction in DMG.^57,58^ ONC201 (dordaviprone), which induces mitochondrial stress, has received Pediatric Rare Disease Designation and is currently in Phase 3 trials for DMG (NCT05580562).^59^ However, ONC201 also activates compensatory PI3K/Akt signaling, promoting metabolic adaptation and resistance.^58,59^ Understanding this metabolic “switch” between glycolysis and oxidative phosphorylation is essential for overcoming resistance in current PI3K/mTOR-targeted trials, including PNOC022 (NCT05009992), where 20% of enrolled patients have survived beyond two years.

#### Pan-PI3K Inhibitors in Clinical Development: Paxalisib and Buparlisib

Paxalisib (formerly GDC-0084) is a BBB-penetrant pan-PI3K inhibitor that inhibits all 4 isoforms of class I PI3K, tested in Phase I and II clinical trials for DMG (Table 1). The therapy was originally developed for the treatment of adult glioblastoma, which frequently harbors PI3K pathway alterations and is hyperactivated in 80% of cases due to deletions in *PTEN*.^60,61^

A Phase I clinical trial for recurrent or progressive HGG (NCT01547546) reported disease stabilization in 40% of the 47 patients, but 11% of patients experienced grade 3 adverse events (AEs), including hyperglycemia and fatigue, with mucositis identified as the primary dose-limiting toxicity (Table 1).^18^ In DMG/DIPG, a Phase I dose-escalation study has been completed in the upfront setting, identifying a maximum tolerated dose (MTD) of 27 mg/m^2^/d (NCT03696355). Our lab discovered paxalisib as a potential therapy for DMG and recently published comprehensive mechanistic studies demonstrating dose optimization and the use of metformin to mitigate hyperglycemia, resulting in improved DMG model survival in vivo.^13,62^

Clinical trials testing paxalisib in DMG, both as monotherapy (NCT03696355) and in combination with ONC201 (NCT05009992), have reported PI3K inhibitor-related side effects, particularly mucositis, rash, colitis, and hyperglycemia. Mucositis is now effectively managed with dexamethasone mouthwash (NCT05009992), but rash remains a challenge. The PI3K/Akt pathway plays a crucial role in insulin signaling, and concurrent corticosteroid therapy, commonly used to manage peritumoral inflammation and hydrocephalus in DMG, further exacerbates glucose dysregulation.

Indeed, treatment with paxalisib at the human-equivalent MTD (10 mg/kg/qd) induced systemic hyperglycemia in DIPG models, consistent with reports in glioblastoma models.^63^ Dose modification, either half MTD daily or twice daily (13.5 mg/m² human equivalent), ameliorated glucose disturbances. Pharmacodynamic analysis of DMG tissues from PDX mice confirmed robust inhibition of pThr308AKT (PI3K) and pSer473AKT (mTOR) at MTD, maintained with half MTD twice daily but once daily.^13^

In efficacy studies, half MTD daily did not improve survival, while MTD extended survival by 10% daily, and half MTD twice daily by 17% compared to vehicle and MTD daily. Co-treatment with metformin (125 mg/kg/qd) further improved survival by 15% (half MTD daily) and 5% (half MTD twice daily), but not at MTD daily, suggesting high-dose PI3K inhibition overrides metformin’s protective effects.^13^ These findings emphasize the need for dose optimization to maximize efficacy while minimizing metabolic toxicity in DMG.

Consistent with our findings, Noch et al.^41^ reported in glioblastoma in vivo models that paxalisib at the adult MTD (15 mg/kg/qd) extended survival, which was further enhanced by metformin (200 mg/kg/qd); combination therapy yielded the most pronounced reductions in p-AKT and p-S6 levels on histological analysis.

Buparlisib (BKM-120) is a BBB-penetrable pan-PI3K inhibitor targeting all 4 class I PI3K isoforms (p110α, p110β, p110δ, and p110γ) and is under clinical development for various brain cancers (Table 1). In glioblastoma, it demonstrated potent anti-migratory effects in vitro and slowed tumor progression in intracranial xenograft models.^64^ However, clinical trials in PI3K-activated recurrent glioblastoma (NCT01339052, Table 1) showed limited benefit, with failure attributed to incomplete downstream PI3K pathway inhibition.^19,41^ Indeed, Noch et al., using data from the Phase 2 study evaluating buparlisib in patients with recurrent GBM (NCT05183204), show that buparlisib activation of insulin signaling, promoting hyperglycemia, was independently associated with poor prognosis.^19^

These challenges are not unique to HGG and DMG. In a clinical trial for brain-metastatic triple-negative breast cancer (NCT01629615), clinical benefit from PI3K inhibition was observed in only 12% of patients, with significant toxicities, including hyperglycemia, rash, and fatigue, similar to those seen with other pan-PI3K inhibitors.^65^ Notably, these studies did not incorporate concurrent anti-glycemia medications.

Immune-related adverse events associated with agents like paxalisib and buparlisib may stem from their pan-PI3K activity. Inhibition of the p110δ isoform, primarily expressed in hematopoietic cells, reduces macrophage recruitment and preferentially suppresses regulatory T cells (Tregs).^33^ While potentially enhancing antitumor immunity, this effect has been linked to hepatotoxicity, colitis, pneumonia, and even intestinal perforation, limiting clinical utility by constraining dosing and drug exposure at the tumor site.

### Novel PIK3CA and mTOR inhibitors: GCT-007 and Compound 7

GCT-007 (Global Cancer Technology) is a brain-penetrant, highly selective inhibitor of p110α, the catalytic subunit of PI3Kα (PIK3CA), making it a promising candidate for HGGs. Unlike pan-PI3K inhibitors, GCT-007 targets the most mutated PI3K isoform in cancers, minimizing off-target effects while maintaining robust antitumor activity. Preclinical studies in glioblastoma have demonstrated that GCT-007 effectively inhibits tumor growth both in vitro and in vivo,^66^ supporting its therapeutic potential for other brain malignancies.

Currently, GCT-007 is under investigation for its efficacy in multiple cancers, including breast cancer and psoriasis, with ongoing research evaluating its utility in DMG within our laboratory. Given its upstream position in the PI3K/Akt/mTOR signaling cascade, GCT-007 offers an opportunity to broadly suppress downstream oncogenic signaling, potentially mitigating compensatory activation of parallel survival pathways. The ability to selectively target PIK3CA may also improve tolerability and reduce the metabolic toxicities commonly associated with pan-PI3K inhibition. Indeed, this selective targeting of PIK3CA has shown more promise than earlier attempts to target downstream AKT directly. While Akt remains a compelling target for cancer therapy, the development of selective inhibitors has been hindered by off-target effects, particularly among other members of the AGC kinase family, including PKA, PKG, and PKC.^67^ ATP-competitive inhibitors often lack sufficient selectivity, prompting interest in alternative approaches. Moreover, the presence of 3 closely related Akt isoforms (Akt1, Akt2, and Akt3), each with emerging and sometimes divergent roles in tumor biology, adds further complexity. Akt1 has been implicated in tumor development and growth, and is primarily regulated via p110α,^68,69^ whereas Akt2 has been associated with tumor invasiveness and metastasis without affecting primary tumor burden.^70,71^ In contrast, higher Akt3 expression has been linked to less aggressive glioblastoma subtypes and improved survival in preclinical models.^72^ The challenge of designing isoform-specific inhibitors due to shared catalytic domain homology has led to the exploration of allosteric inhibitors and other strategies that may enable more precise modulation of Akt signaling.^73^

Compound 7 (Novartis) is a highly BBB-penetrable dual inhibitor of both mTOR complexes (mTORC1 and mTORC2).^74^ Among PI3K pathway inhibitors, BBB permeability remains a critical challenge with most mTOR inhibitors failing to effectively reach the tumor sites in the CNS (Table 2). Aside from Compound 7, PQR620 is the only other reported selective dual mTORC1/mTORC2 inhibitor with demonstrated BBB penetrance.^75^

Beyond its potential application in cancer, Compound 7 has shown promising results in models of neurological disorders, highlighting its role in regulating mTOR-dependent neurodevelopmental and neurodegenerative processes.^75^ Notably, in neuronal cell-based models, Compound 7 successfully inhibited mTOR signaling and extended the survival of murine models harboring neuron-specific loss of *Tsc1*, a negative regulator of mTOR.^74^

While direct inhibition of mTOR presents a compelling strategy to block PI3K/Akt/mTOR signaling, it is important to consider whether targeting PIK3CA upstream may offer a more effective therapeutic approach. Inhibiting p110α at the top of the cascade may prevent compensatory activation of alternative survival mechanisms downstream. Given that mTOR inhibition alone does not address PI3K-driven hyperglycemia, a key metabolic challenge in PI3K-targeted therapies, selective p110α inhibitors like GCT-007 may provide a more integrated approach to pathway suppression in DMG. Future studies should assess the comparative efficacy of p110α and dual mTORC1/2 inhibition in vivo to determine the most effective strategy for suppressing PI3K pathway activity while minimizing resistance.

### PI3K/mTOR Inhibition Improves Immunotherapy Efficacy

**Figure 4.**
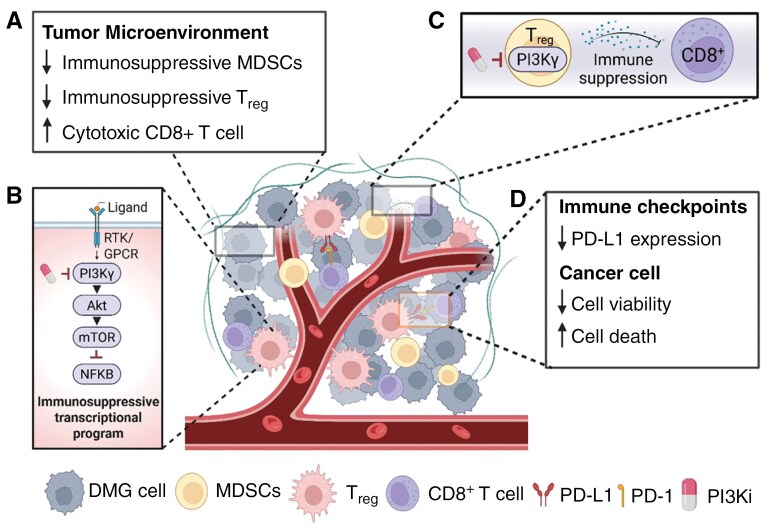
PI3K/Akt/mTOR inhibition enhances antitumor immune responses. (A) Regulatory T cells (Tregs) suppress effective antitumor immunity. Selective inhibition of PI3Kγ reduces Treg abundance, correlating with increased infiltration of CD8⁺ T cells expressing activation markers such as granzyme B.^77–79^ (B) In myeloid-derived suppressor cells (MDSCs), PI3Kγ activation suppresses NF-κB signaling via Akt/mTOR, driving an immunosuppressive transcriptional program.^77^ (C) Inactivation of PI3Kγ in MDSCs reverses this suppression, promoting an immunostimulatory phenotype that restores CD8⁺ T cell activity and cytotoxicity.^78^ (D) PI3K/Akt/mTOR signaling also regulates expression of immune checkpoints such as anti-programmed death protein 1 (anti-PD-1) in the tumor but also in tumor-associated macrophages (TAMs) by epigenetic mechanisms.^76^ (Created in BioRender. Dun (2025) https://BioRender.com/df1ozvh).

Emerging evidence suggests that inhibition of the PI3K/mTOR pathway enhances the efficacy of immunotherapy through multiple mechanisms, including modulation of the tumor microenvironment, epigenetic reprogramming, and direct effects on immune cell function.^76^ PI3K/mTOR signaling plays a key role in regulating the expression of immune checkpoints such as PD-L1, with inhibition shown to downregulate PD-L1 expression via epigenetic mechanisms including histone deacetylation and methylation changes (Figure 4).^77,80,81^

In preclinical and clinical studies, inhibition of PI3K (particularly PI3Kγ and PI3Kδ in myeloid and lymphoid cells, respectively) promotes the infiltration and activation of cytotoxic CD8+ T cells while reducing immunosuppressive regulatory T cells and myeloid-derived suppressor cells (Figure 4).^77–79^

Furthermore, culturing chimeric antigen receptor (CAR) T cells in the presence of PI3K or mTOR inhibitors during ex vivo expansion can improve their metabolic fitness, persistence, and antitumor efficacy upon infusion. Agents such as idelalisib, rapamycin, and metformin, the latter activating TSC1/2 through AMPK and thereby inhibiting mTORC1,^82^ have been shown to promote the development of memory-like T cells with greater in vivo persistence and cytotoxicity.^83–85^

However, the timing and dosing of PI3K/mTOR inhibition are critical. Excessive inhibition, particularly systemic or sustained high-dose administration, can lead to immunosuppression by impairing T-cell proliferation and survival, effectively negating the benefits of immunotherapy.^13,86^ This has been demonstrated in our own studies optimizing pharmacokinetics (PK) and pharmacodynamics (PD) of PI3K/mTOR-targeted therapies in DMG, where we observed a narrow therapeutic window. Thus, careful balance is required to enhance immune response without compromising immune cell viability.^13^

Given the immunologically “cold” nature of pediatric HGGs and DMGs—characterized by low levels of T cells—rationally combining the immunostimulatory effects of PI3K/mTOR inhibitors with ICIs or CAR T cells may represent a critical avenue to overcome resistance and drive more durable antitumor responses. Future studies should focus on defining optimal schedules, dosing, and patient selection to maximize the synergistic potential of these multimodal strategies.

## Discussion

PI3K and mTOR represent clear genetic dependencies in pHGG, including DMGs. Recent advances in the development of brain-penetrant pan- and selective PI3K and mTOR inhibitors provide the field with potent new weapons against these historically lethal pediatric brain cancers. However, despite these promising tools, there remains substantial work to do if we are to meaningfully improve patient outcomes.

Here, we have outlined the advantages and limitations of both pan- and isoform-selective PI3K/mTOR inhibitors that can reach the brain. Selective inhibitors may reduce systemic toxicity and enhance tumor-targeted efficacy, whereas pan-inhibitors, while potentially more toxic, may yield superior responses when dosed optimally or combined with immunotherapies. Emerging studies support the potential of both approaches, but as a field, we must take greater care to interpret and apply these insights if we are to move forward effectively.

The highly adaptive nature of the DMG epigenome, driven by hallmark H3-alterations, necessitates rational combination strategies to overcome therapeutic resistance. Given the tumor’s capacity to activate compensatory survival pathways, single-agent PI3K inhibitors are unlikely to produce durable responses. Instead, integrating PI3K/mTOR inhibition with complementary therapies that target parallel or downstream mechanisms may hold greater promise for long-term control.

The path forward, moving PI3K/mTOR inhibitors from research and early clinical development and then toward regulatory approval for pHGGs, is complex. We must not only improve therapeutic outcomes but also ensure that this endeavor remains viable and attractive for researchers and industry partners. This means optimizing therapeutic windows, selecting synergistic drug combinations, managing toxicities, and aligning treatment with immunotherapy strategies. Equally important is identifying the patients most likely to respond.

We remain hopeful that, over the coming years, these challenges will be addressed. With sustained collaboration and innovation, we can climb beyond the preclinical *Base Camp* and begin the final ascent toward the summit of the *most lethal mountain* in pediatric oncology-DMG.

## References

[1] Mackay A, Burford A, Carvalho D, et al Integrated molecular meta-analysis of 1,000 pediatric high-grade and diffuse intrinsic pontine glioma. Cancer Cell. 2017;32(4):520–537.e5. 28966033 10.1016/j.ccell.2017.08.017 PMC5637314

[2] Panizo-Morgado E, Vazquez-Gómez F, Perez-Somarriba M, et al Re-irradiation for progressive diffuse intrinsic pontine glioma (DIPG): the Spanish experience. EJC Paediatric Oncol. 2024;4:100183.

[3] Shariff N, Moreno AS, Bennett J, et al Re-irradiation for children with diffuse intrinsic pontine glioma and diffuse midline glioma. Radiother Oncol. 2025;207:110865. 40139463 10.1016/j.radonc.2025.110865

[4] Duchatel RJ, Jackson ER, Alvaro F, et al Signal transduction in diffuse intrinsic pontine glioma. Proteomics. 2019;19(21-22):e1800479. 31328874 10.1002/pmic.201800479

[5] Findlay IJ, De Iuliis GN, Duchatel RJ, et al Pharmaco-proteogenomic profiling of pediatric diffuse midline glioma to inform future treatment strategies. Oncogene. 2022;41(4):461–475. 34759345 10.1038/s41388-021-02102-y PMC8782719

[6] Arms LM, Duchatel RJ, Jackson ER, et al Current status and advances to improving drug delivery in diffuse intrinsic pontine glioma. J Control Release. 2024;370:835–865. 38744345 10.1016/j.jconrel.2024.05.018

[7] Persson ML, Douglas AM, Alvaro F, et al The intrinsic and microenvironmental features of diffuse midline glioma: implications for the development of effective immunotherapeutic treatment strategies. Neuro-Oncology. 2022;24(9):1408–1422. 35481923 10.1093/neuonc/noac117 PMC9435509

[8] Katso R, Okkenhaug K, Ahmadi K, et al Cellular function of phosphoinositide 3-kinases: implications for development, homeostasis, and cancer. Annu Rev Cell Dev Biol. 2001;17(1):615–675. 11687500 10.1146/annurev.cellbio.17.1.615

[9] Engelman JA, Luo J, Cantley LC. The evolution of phosphatidylinositol 3-kinases as regulators of growth and metabolism. Nat Rev Genet. 2006;7(8):606–619. 16847462 10.1038/nrg1879

[10] Yang J, Nie J, Ma X, et al Targeting PI3K in cancer: mechanisms and advances in clinical trials. Mol Cancer. 2019;18(1):26. 30782187 10.1186/s12943-019-0954-x PMC6379961

[11] Jean S, Kiger AA. Classes of phosphoinositide 3-kinases at a glance. J Cell Sci. 2014;127(5):923–928. 10.1242/jcs.093773 PMC3937771 24587488

[12] Fuso P, Muratore M, D’Angelo T, et al PI3K inhibitors in advanced breast cancer: the past, the present, new challenges and future perspectives. Cancers. 2022;14(9):2161. 35565291 10.3390/cancers14092161 PMC9103982

[13] Duchatel RJ, Jackson ER, Parackal SG, et al PI3K/mTOR is a therapeutically targetable genetic dependency in diffuse intrinsic pontine glioma. J Clin Invest. 2024;134(6):e170329. 38319732 10.1172/JCI170329 PMC10940093

[14] Goncalves MD , et al Phosphatidylinositol 3-kinase, growth disorders, and cancer. N Engl J Med. 2018;379(21):2052–2062. 30462943 10.1056/NEJMra1704560

[15] Mizoguchi M, Nutt CL, Mohapatra G, Louis DN. Genetic alterations of phosphoinositide 3-kinase subunit genes in human glioblastomas. Brain Pathol. 2004;14(4):372–377. 15605984 10.1111/j.1750-3639.2004.tb00080.x PMC8095817

[16] Chakravarti A, Zhai G, Suzuki Y, et al The prognostic significance of phosphatidylinositol 3-kinase pathway activation in human gliomas. J Clin Oncol. 2004;22(10):1926–1933. 10.1200/JCO.2004.07.193 15143086

[17] Tinkle C, Huang J, Campagne O, et al CTNI-27. first-in-pediatrics phase I study of GDC-0084 (Paxalisib): a CNS-penetrant PI3K/mTOR inhibitor, in newly diagnosed diffuse intrinsic pontine glioma (DIPG) or other diffuse midline glioma (DMG). Neuro-Oncology. 2020;22(Suppl_2):ii48–ii48.

[18] Wen PY, Cloughesy TF, Olivero AG, et al First-in-human phase I study to evaluate the brain-penetrant PI3K/mTOR inhibitor GDC-0084 in patients with progressive or recurrent high-grade glioma. Clin Cancer Res. 2020;26(8):1820–1828. 31937616 10.1158/1078-0432.CCR-19-2808

[19] Wen PY, Touat M, Alexander BM, et al Buparlisib in patients with recurrent glioblastoma harboring phosphatidylinositol 3-kinase pathway activation: an open-label, multicenter, multi-arm, phase II trial. J Clin Oncol. 2019;37(9):741–750. 30715997 10.1200/JCO.18.01207 PMC6553812

[20] van den Bent M, Azaro A, De Vos F, et al A Phase Ib/II, open-label, multicenter study of INC280 (capmatinib) alone and in combination with buparlisib (BKM120) in adult patients with recurrent glioblastoma. J Neurooncol. 2020;146(1):79–89. 31776899 10.1007/s11060-019-03337-2 PMC6938467

[21] Wen PY, Rodon JA, Mason W, et al Phase I, open-label, multicentre study of buparlisib in combination with temozolomide or with concomitant radiation therapy and temozolomide in patients with newly diagnosed glioblastoma. ESMO Open. 2020;5(4):e000673. 32661186 10.1136/esmoopen-2020-000673 PMC7359189

[22] Shih KC, Chowdhary SA, Becker KP, et al A phase II study of the combination of BKM120 (buparlisib) and bevacizumab in patients with relapsed/refractory glioblastoma multiforme (GBM). J Clin Oncol. 2015;33(15_suppl):2065–2065.

[23] Janku F, Yap TA, Meric-Bernstam F. Targeting the PI3K pathway in cancer: are we making headway? Nat Rev Clin Oncol. 2018;15(5):273–291. 29508857 10.1038/nrclinonc.2018.28

[24] Rodon J, Dienstmann R, Serra V, Tabernero J. Development of PI3K inhibitors: lessons learned from early clinical trials. Nat Rev Clin Oncol. 2013;10(3):143–153. 23400000 10.1038/nrclinonc.2013.10

[25] Chen S, Liu Y, Rong X, et al Neuroprotective role of the PI3 kinase/Akt signaling pathway in Zebrafish. Front Endocrinol (Lausanne). 2017;8:21. 28228749 10.3389/fendo.2017.00021 PMC5296330

[26] Keppler-Noreuil KM , et al Clinical delineation and natural history of the PIK3CA-related overgrowth spectrum. Am J Med Genet A. 2014;164A(7):1713–1733. 24782230 10.1002/ajmg.a.36552 PMC4320693

[27] Jansen LA, Mirzaa GM, Ishak GE, et al PI3K/AKT pathway mutations cause a spectrum of brain malformations from megalencephaly to focal cortical dysplasia. Brain. 2015;138(6):1613–1628. 25722288 10.1093/brain/awv045 PMC4614119

[28] Roy A, Skibo J, Kalume F, et al Mouse models of human PIK3CA-related brain overgrowth have acutely treatable epilepsy. eLife. 2015;4:e12703. 10.7554/eLife.12703 PMC4744197 26633882

[29] Vyas P, Tulsawani R, Vohora D. Dual targeting by inhibition of phosphoinositide-3-kinase and mammalian target of rapamycin attenuates the neuroinflammatory responses in murine hippocampal cells and seizures in C57BL/6 mice. Front Immunol. 2021;12:739452. 34887852 10.3389/fimmu.2021.739452 PMC8650161

[30] Levine DA, Bogomolniy F, Yee CJ, et al Frequent mutation of the PIK3CA gene in ovarian and breast cancers. Clin Cancer Res. 2005;11(8):2875–2878. 15837735 10.1158/1078-0432.CCR-04-2142

[31] Hayes MP, Wang H, Espinal-Witter R, et al PIK3CA and PTEN mutations in uterine endometrioid carcinoma and complex atypical hyperplasia. Clin Cancer Res. 2006;12(20):5932–5935. 17062663 10.1158/1078-0432.CCR-06-1375

[32] Sanaei MJ, Razi S, Pourbagheri-Sigaroodi A, Bashash D. The PI3K/Akt/mTOR pathway in lung cancer; oncogenic alterations, therapeutic opportunities, challenges, and a glance at the application of nanoparticles. Transl Oncol. 2022;18:101364. 35168143 10.1016/j.tranon.2022.101364 PMC8850794

[33] Yu M, Chen J, Xu Z, et al Development and safety of PI3K inhibitors in cancer. Arch Toxicol. 2023;97(3):635–650. 36773078 10.1007/s00204-023-03440-4 PMC9968701

[34] Andre F , et al Alpelisib for PIK3CA-mutated, hormone receptor-positive advanced breast cancer. N Engl J Med. 2019;380(20):1929–1940. 31091374 10.1056/NEJMoa1813904

[35] Markham AA. First global approval. Drugs. 2019;79(11):1249–1253. 31256368 10.1007/s40265-019-01161-6

[36] Patel K, Danilov AV, Pagel JM. Duvelisib for CLL/SLL and follicular non-Hodgkin lymphoma. Blood. 2019;134(19):1573–1577. 31554637 10.1182/blood.2019001795 PMC9635582

[37] Fowler NH, Samaniego F, Jurczak W, et al Umbralisib, a dual PI3Kdelta/CK1epsilon inhibitor in patients with relapsed or refractory indolent lymphoma. J Clin Oncol. 2021;39(15):1609–1618. 33683917 10.1200/JCO.20.03433 PMC8148421

[38] Pavel ME, Hainsworth JD, Baudin E, et al Everolimus plus octreotide long-acting repeatable for the treatment of advanced neuroendocrine tumours associated with carcinoid syndrome (RADIANT-2): a randomised, placebo-controlled, phase 3 study. Lancet. 2011;378(9808):2005–2012. 22119496 10.1016/S0140-6736(11)61742-X

[39] Hess G, Herbrecht R, Romaguera J, et al Phase III study to evaluate temsirolimus compared with investigator’s choice therapy for the treatment of relapsed or refractory mantle cell lymphoma. J Clin Oncol. 2009;27(23):3822–3829. 19581539 10.1200/JCO.2008.20.7977

[40] Wager TT, Hou X, Verhoest PR, Villalobos A. Central nervous system multiparameter optimization desirability: application in drug discovery. ACS Chem Neurosci. 2016;7(6):767–775. 26991242 10.1021/acschemneuro.6b00029

[41] Noch EK, Palma LN, Yim I, et al Insulin feedback is a targetable resistance mechanism of PI3K inhibition in glioblastoma. Neuro-Oncology. 2023;25(12):2165–2176. 37399061 10.1093/neuonc/noad117 PMC10708938

[42] Huang X , et al The PI3K/AKT pathway in obesity and type 2 diabetes. Int J Biol Sci. 2018;14(11):1483–1496. 30263000 10.7150/ijbs.27173 PMC6158718

[43] Hanker AB, Kaklamani V, Arteaga CL. Challenges for the clinical development of PI3K inhibitors: strategies to improve their impact in solid tumors. Cancer Discov. 2019;9(4):482–491. 30867161 10.1158/2159-8290.CD-18-1175 PMC6445714

[44] Hopkins BD, Goncalves MD, Cantley LC. Insulin-PI3K signalling: an evolutionarily insulated metabolic driver of cancer. Nat Rev Endocrinol. 2020;16(5):276–283. 32127696 10.1038/s41574-020-0329-9 PMC7286536

[45] Moore HN, Goncalves MD, Johnston AM, et al Effective strategies for the prevention and mitigation of phosphatidylinositol-3-kinase inhibitor-associated hyperglycemia: optimizing patient care. Clin Breast Cancer. 2025;25(1):1–11. 39462728 10.1016/j.clbc.2024.09.017

[46] He C, Xu K, Zhu X, et al Patient-derived models recapitulate heterogeneity of molecular signatures and drug response in pediatric high-grade glioma. Nat Commun. 2021;12(1):4089. 34215733 10.1038/s41467-021-24168-8 PMC8253809

[47] Minichiello L. TrkB signalling pathways in LTP and learning. Nat Rev Neurosci. 2009;10(12):850–860. 19927149 10.1038/nrn2738

[48] Taylor KR, Barron T, Hui A, et al Glioma synapses recruit mechanisms of adaptive plasticity. Nature. 2023;623(7986):366–374. 37914930 10.1038/s41586-023-06678-1 PMC10632140

[49] Geraldes P, King GL. Activation of protein kinase C isoforms and its impact on diabetic complications. Circ Res. 2010;106(8):1319–1331. 20431074 10.1161/CIRCRESAHA.110.217117 PMC2877591

[50] Park E, Chen J, Moore A, et al Stromal cell protein kinase C-beta inhibition enhances chemosensitivity in B cell malignancies and overcomes drug resistance. Sci Transl Med. 2020;12(526):eaax9340. 31941829 10.1126/scitranslmed.aax9340 PMC7116365

[51] Hurtado E, Cilleros V, Nadal L, et al Muscle contraction regulates BDNF/TrkB signaling to modulate synaptic function through presynaptic cPKCalpha and cPKCbetaI. Front Mol Neurosci. 2017;10:147. 28572757 10.3389/fnmol.2017.00147 PMC5436293

[52] Obis T, Hurtado E, Nadal L, et al The novel protein kinase C epsilon isoform modulates acetylcholine release in the rat neuromuscular junction. Mol Brain. 2015;8(1):80. 26625935 10.1186/s13041-015-0171-5 PMC4665914

[53] Murray HC, Enjeti AK, Kahl RGS, et al Quantitative phosphoproteomics uncovers synergy between DNA-PK and FLT3 inhibitors in acute myeloid leukaemia. Leukemia. 2021;35(6):1782–1787. 33067575 10.1038/s41375-020-01050-y PMC8179851

[54] Murray HC, Miller K, Brzozowski JS, et al Synergistic targeting of DNA-PK and KIT signaling pathways in KIT mutant acute myeloid leukemia. Mol Cell Proteomics. 2023;22(3):100503. 36682716 10.1016/j.mcpro.2023.100503 PMC9986649

[55] Lang F, Liu Y, Chou F-J, Yang C. Genotoxic therapy and resistance mechanism in gliomas. Pharmacol Ther. 2021;228:107922. 34171339 10.1016/j.pharmthera.2021.107922 PMC8848306

[56] Gil del Alcazar CR, Hardebeck MC, Mukherjee B, et al Inhibition of DNA double-strand break repair by the dual PI3K/mTOR inhibitor NVP-BEZ235 as a strategy for radiosensitization of glioblastoma. Clin Cancer Res. 2014;20(5):1235–1248. 24366691 10.1158/1078-0432.CCR-13-1607 PMC3947495

[57] Przystal JM, Cianciolo Cosentino C, Yadavilli S, et al Imipridones affect tumor bioenergetics and promote cell lineage differentiation in diffuse midline gliomas. Neuro-Oncology. 2022;24(9):1438–1451. 35157764 10.1093/neuonc/noac041 PMC9435508

[58] Jackson ER, Persson ML, Fish CJ, et al A review of current therapeutics targeting the mitochondrial protease ClpP in diffuse midline glioma, H3 K27-altered. Neuro-Oncology. 2024;26(Suppl_2):S136–S154. 37589388 10.1093/neuonc/noad144 PMC11066926

[59] Jackson ER, Duchatel RJ, Staudt DE, et al ONC201 in combination with paxalisib for the treatment of H3K27-altered diffuse midline glioma. Cancer Res. 2023;83(14):2421–2437.37195023 10.1158/0008-5472.CAN-23-0186

[60] Mueller S, Phillips J, Onar-Thomas A, et al PTEN promoter methylation and activation of the PI3K/Akt/mTOR pathway in pediatric gliomas and influence on clinical outcome. Neuro-Oncology. 2012;14(9):1146–1152. 22753230 10.1093/neuonc/nos140 PMC3424210

[61] Brennan CW , et al The somatic genomic landscape of glioblastoma. Cell. 2013;155(2):462–477. 24120142 10.1016/j.cell.2013.09.034 PMC3910500

[62] Tzaridis T, Wechsler-Reya RJ. Just a spoonful of metformin helps the medicine go down. J Clin Invest. 2024;134(6):e179144. 38488006 10.1172/JCI179144 PMC10940077

[63] Hopkins BD, Pauli C, Du X, et al Suppression of insulin feedback enhances the efficacy of PI3K inhibitors. Nature. 2018;560(7719):499–503. 30051890 10.1038/s41586-018-0343-4 PMC6197057

[64] Speranza MC, Nowicki MO, Behera P, et al BKM-120 (Buparlisib): a phosphatidyl-inositol-3 kinase inhibitor with anti-invasive properties in glioblastoma. Sci Rep. 2016;6(1):20189. 26846842 10.1038/srep20189 PMC4742861

[65] Garrido-Castro AC, Saura C, Barroso-Sousa R, et al Phase 2 study of buparlisib (BKM120), a pan-class I PI3K inhibitor, in patients with metastatic triple-negative breast cancer. Breast Cancer Res. 2020;22(1):120. 33138866 10.1186/s13058-020-01354-y PMC7607628

[66] Technology GC. GCT-007: Progress and Pipeline. 2022; https://globalcancertechnology.com/pipeline/. Accessed 21 March 2024.

[67] Nitulescu GM, Margina D, Juzenas P, et al Akt inhibitors in cancer treatment: the long journey from drug discovery to clinical use (Review). Int J Oncol. 2016;48(3):869–885. 26698230 10.3892/ijo.2015.3306 PMC4750533

[68] Hutchinson JN, Jin J, Cardiff RD, Woodgett JR, Muller WJ. Activation of Akt-1 (PKB-α) can accelerate ErbB-2-mediated mammary tumorigenesis but suppresses tumor invasion. Cancer Res. 2004;64(9):3171–3178. 15126356 10.1158/0008-5472.can-03-3465

[69] Matheny RW, Adamo ML. PI3K p110α and p110β have differential effects on Akt activation and protection against oxidative stress-induced apoptosis in myoblasts. Cell Death Differ. 2010;17(4):677–688. 19834495 10.1038/cdd.2009.150 PMC2839024

[70] Dillon RL, Marcotte R, Hennessy BT, et al Akt1 and Akt2 play distinct roles in the initiation and metastatic phases of mammary tumor progression. Cancer Res. 2009;69(12):5057–5064. 19491266 10.1158/0008-5472.CAN-08-4287 PMC4151524

[71] Degtyarev M, De Mazière A, Orr C, et al Akt inhibition promotes autophagy and sensitizes PTEN-null tumors to lysosomotropic agents. J Cell Biol. 2008;183(1):101–116. 18838554 10.1083/jcb.200801099 PMC2557046

[72] Joy A, Kapoor M, Georges J, et al The role of AKT isoforms in glioblastoma: AKT3 delays tumor progression. J Neurooncol. 2016;130(1):43–52. 27422127 10.1007/s11060-016-2220-z PMC5071179

[73] Mattmann ME, Stoops SL, Lindsley CW. Inhibition of Akt with small molecules and biologics: historical perspective and current status of the patent landscape. Expert Opin Ther Pat. 2011;21(9):1309–1338. 21635152 10.1517/13543776.2011.587959 PMC4279453

[74] Bonazzi S , et al Discovery of a brain-penetrant ATP-competitive inhibitor of the mechanistic target of rapamycin (mTOR) for CNS disorders. J Med Chem. 2020;63(3):1068–1083. 31955578 10.1021/acs.jmedchem.9b01398

[75] Rageot D , et al Discovery and preclinical characterization of 5-[4,6-Bis(3-oxa-8-azabicyclo[3.2.1]octan-8-yl)-1,3,5-triazin-2-yl]-4-(difluoromethyl)pyridin-2-amine (PQR620), a highly potent and selective mTORC1/2 inhibitor for cancer and neurological disorders. J Med Chem. 2018;61(22):10084–10105. 30359003 10.1021/acs.jmedchem.8b01262

[76] Zhang H, Liu L, Liu J, et al Roles of tumor-associated macrophages in anti-PD-1/PD-L1 immunotherapy for solid cancers. Mol Cancer. 2023;22(1):58. 36941614 10.1186/s12943-023-01725-x PMC10029244

[77] Kaneda MM, Messer KS, Ralainirina N, et al PI3Kgamma is a molecular switch that controls immune suppression. Nature. 2016;539(7629):437–442. 27642729 10.1038/nature19834 PMC5479689

[78] De Henau O, Rausch M, Winkler D, et al Overcoming resistance to checkpoint blockade therapy by targeting PI3Kgamma in myeloid cells. Nature. 2016;539(7629):443–447. 27828943 10.1038/nature20554 PMC5634331

[79] Ali K, Soond DR, Piñeiro R, et al Inactivation of PI(3)K p110delta breaks regulatory T-cell-mediated immune tolerance to cancer. Nature. 2014;510(7505):407–411. 24919154 10.1038/nature13444 PMC4501086

[80] Lastwika KJ, Wilson W, Li QK, et al Control of PD-L1 expression by oncogenic activation of the AKT-mTOR pathway in non-small cell lung cancer. Cancer Res. 2016;76(2):227–238. 26637667 10.1158/0008-5472.CAN-14-3362

[81] Peng W , et al Loss of PTEN promotes resistance to T cell-mediated immunotherapy. Cancer Discov. 2016;6(2):202–216. 26645196 10.1158/2159-8290.CD-15-0283 PMC4744499

[82] Howell JJ, Hellberg K, Turner M, et al Metformin inhibits hepatic mTORC1 signaling via dose-dependent mechanisms involving AMPK and the TSC complex. Cell Metab. 2017;25(2):463–471. 28089566 10.1016/j.cmet.2016.12.009 PMC5299044

[83] Crompton JG, Sukumar M, Roychoudhuri R, et al Akt inhibition enhances expansion of potent tumor-specific lymphocytes with memory cell characteristics. Cancer Res. 2015;75(2):296–305. 25432172 10.1158/0008-5472.CAN-14-2277 PMC4384335

[84] Kawalekar OU, O’Connor RS, Fraietta JA, et al Distinct signaling of coreceptors regulates specific metabolism pathways and impacts memory development in CAR T cells. Immunity. 2016;44(2):380–390. 26885860 10.1016/j.immuni.2016.01.021 PMC13498396

[85] Hatae R, Kyewalabye K, Yamamichi A, et al Enhancing CAR-T cell metabolism to overcome hypoxic conditions in the brain tumor microenvironment. JCI Insight. 2024;9(7):e177141. 38386420 10.1172/jci.insight.177141 PMC11128202

[86] Ahmadzadeh M, Johnson LA, Heemskerk B, et al Tumor antigen-specific CD8 T cells infiltrating the tumor express high levels of PD-1 and are functionally impaired. Blood. 2009;114(8):1537–1544. 19423728 10.1182/blood-2008-12-195792 PMC2927090

